# First person – Destynie Medeiros

**DOI:** 10.1242/dmm.050803

**Published:** 2024-05-24

## Abstract

First Person is a series of interviews with the first authors of a selection of papers published in Disease Models & Mechanisms, helping researchers promote themselves alongside their papers. Destynie Medeiros is first author on ‘ A small-molecule TrkB ligand improves dendritic spine phenotypes and atypical behaviors in female Rett syndrome mice’, published in DMM. Destynie is a PhD student in the lab of Lucas Pozzo-Miller at the University of Alabama at Birmingham, USA, investigating the dendritic spine and social behavior phenotypes in mouse models for Rett syndrome.


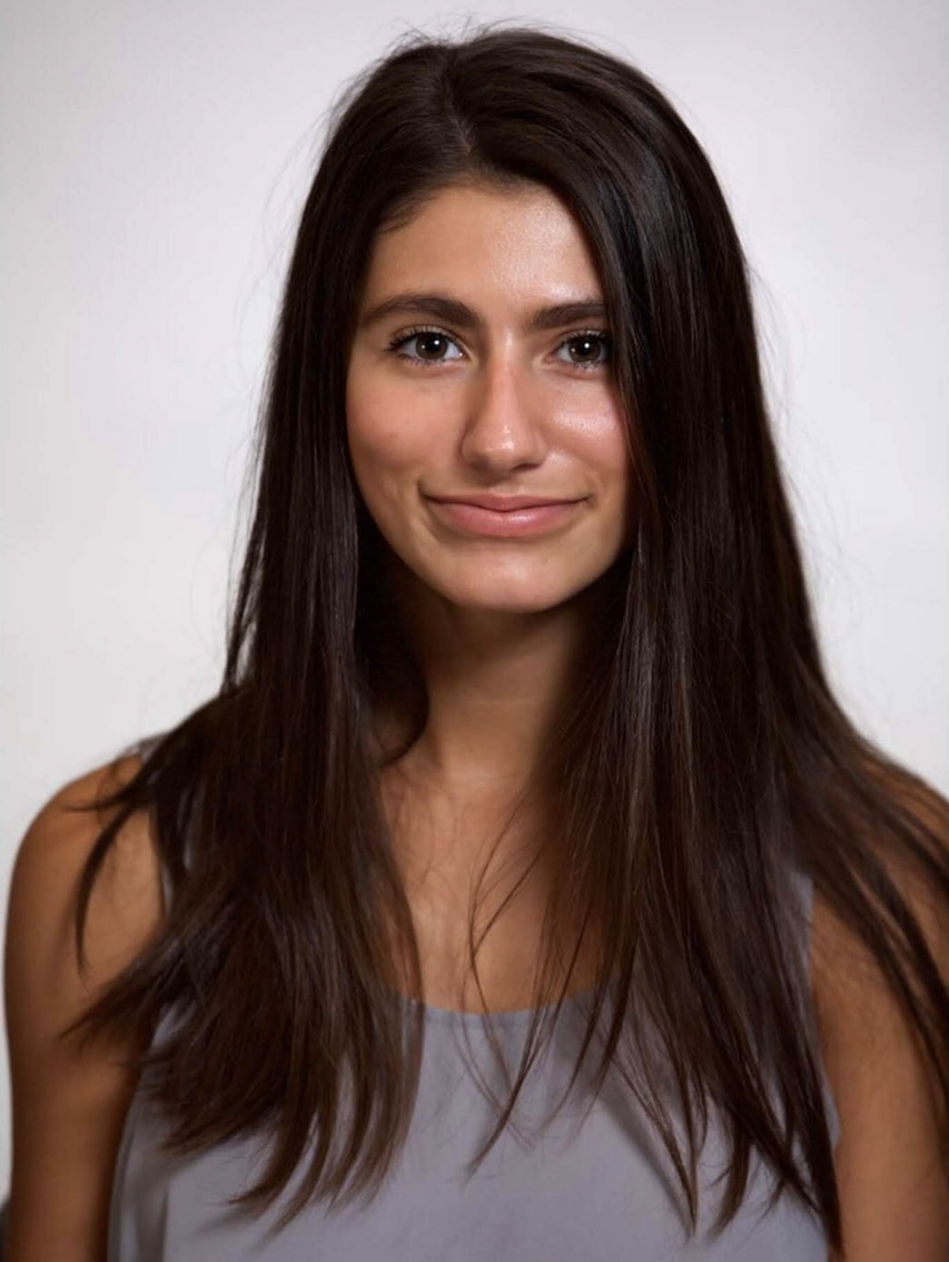


**Destynie Medeiros**

**How would you explain the main findings of your paper to non-scientific family and friends?**

Our work serves to further our understanding of a neurodevelopmental, autism-associated disorder, Rett syndrome, that primarily affects females as it is caused by mutations in the X chromosome. As females have two X chromosomes, in a female Rett brain, about half of the cells will be mutant, whereas the other half will be healthy. We found that by using a female mouse model for Rett syndrome, we were able to uncover a novel alteration in how mutant neurons interact with healthy cells in the brain through protrusions known as ‘dendritic spines’. We found that in the female Rett brain, the healthy cells were the population that appeared larger than healthy cells, which was reminiscent to us of our findings in male Rett mice. All the while, mutant cells presented as healthy and smaller, suggesting that these mutant cells are affecting the function of healthy cells. To combat this phenotype, we utilized a drug known as LM22A-4 to improve the growth and health of neurons and we found that this drug treatment improved the dendritic spine aberration we observed and reduced the size of dendritic spines of healthy neurons in the female Rett brain. We also looked at the social behaviors of these female Rett mice, focusing on social preference, social memory and naturalistic behaviors. We observed that female Rett mice displayed enhanced aggression towards other mice, which were normalized when mice were treated with LM22A-4. Our results suggest that neuronal dysfunction in Rett syndrome is far more complex in the female mouse brain, in which healthy neurons can be affected by the mutant neurons. We also identified a novel aggression phenotype that is largely unexplored in female mice.

Our results suggest that neuronal dysfunction in Rett syndrome is far more complex in the female mouse brain, in which healthy neurons can be affected by the mutant neurons.

**What are the potential implications of these results for your field of research?**

Rett syndrome is a rare, debilitating neurodevelopmental disorder that primarily affects females, and leads to severe mental and physical disability. Treatment options for Rett syndrome are limited and often are supportive by targeting symptoms. Our study utilized a drug compound known as LM22A-4, which targets brain-derived neurotrophic factor (BDNF) signaling as a therapeutic approach in Rett syndrome mouse models. Our findings support the usefulness of BDNF-related treatments for neurodevelopmental disorders such as Rett syndrome.

**What are the main advantages and drawbacks of the experimental system you have used as it relates to the disease you are investigating?**

Our work utilizes mouse models to study Rett syndrome, a genetic neurodevelopmental disorder. This allows for genetic similarities as mice can be used to mimic the genetic mutations seen in individuals with Rett syndrome, and thus provide a live organism to study disease mechanisms and progression. Mice allow for detailed behavioral and biochemical studies within a controlled environment to minimize confounding variables. The use of mice for preclinical development of novel drugs or treatment strategies is ideal for efficacy and safety prior to clinical trials. All the while, as mice have a relatively short lifespan in comparison to humans, we can study disease progression to understand long-term deficits and outcomes. However, working with a mouse model system also presents with certain limitations, including species differences and model limitations of studying a human disorder in a mouse model, which cannot and will not recapitulate all aspects of the condition in individuals living with Rett syndrome. Furthermore, mice are behaviorally complex and we can interpret mouse behavior data only as far as our testing allows us; however, this cannot replicate the complexity of human behavior. It is necessary to understand the sacrifice of using animals in biomedical research, which requires emphasis on reducing animal use and finding and using alternative research models.

**What has surprised you the most while conducting your research?**

The progression and complexity of mouse behaviors! We found that at 5 months of age, female Rett mice engaged in aggressive behaviors more than wildtype female mice; however, this effect was absent in a separate group of mice at 7 months of age, where we found instead that female Rett mice displayed atypical ‘shuffling’ locomotion. These results highlight that the progression of behavioral phenotypes in female Rett mice is not a simple process of all deficits equally worsening with age, resembling the onset and waning of different neuropsychiatric signs in individuals with Rett syndrome throughout various phases of the disorder.

**Figure:**
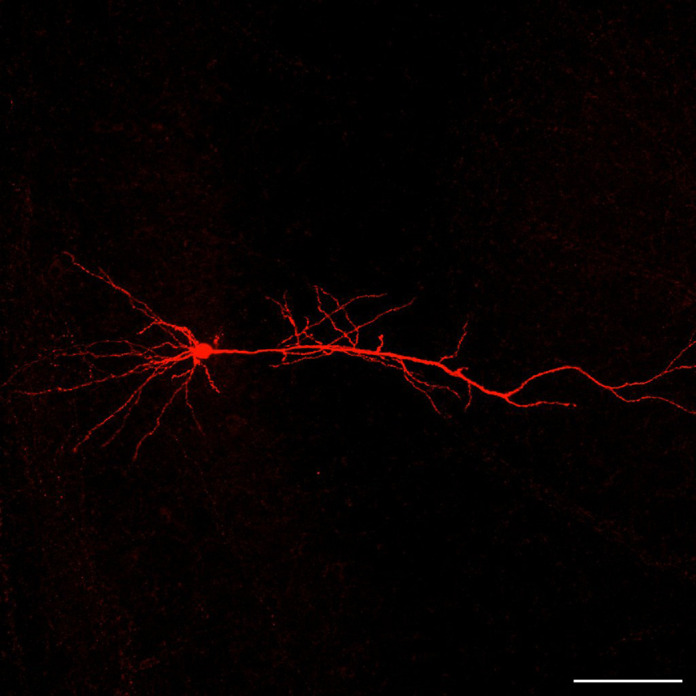
**Biocytin-filled hippocampal CA1 pyramidal neuron in a slice from a female Rett syndrome mouse.** Scale bar: 100 µm.

**What do you think is the most significant challenge impacting your research at this time and how will this be addressed over the next 10 years?**

The most significant challenge in my research I believe is the need for a deeper understanding of rodent behaviors, which will be addressed by advancing machine learning approaches for analyzing behavioral phenotypes. This includes studying more naturalistic and complex behaviors, complemented by targeting specific neuronal circuits associated with these behaviors. Another challenge is the *in vivo* imaging of dendritic spines to gain a functional understanding of their dynamic alterations in disease. Overcoming the hurdles of managing large datasets and the high costs associated with this research will be crucial in the next decade.

**What is one thing you would highlight that was important for the success of your research?**

A key element in the success of my research has been the laboratory environment. The collaborative spirit within the Pozzo-Miller lab was instrumental. This project was a team effort, made possible by a group of dedicated individuals who share a commitment to both scientific inquiry and the growth of trainees. This nurturing atmosphere, where asking questions and supporting each other's learning goes hand in hand, has been invaluable throughout my doctoral training.

Having Dr Lucas Pozzo-Miller as a mentor has been particularly crucial for my development and aspirations towards an academic career. His approach to teaching, combined with genuine encouragement and support, has been pivotal in driving my research and shaping my personal growth.

Real commitment to diversity, equity and inclusion means transforming our workplaces into environments where diverse perspectives fuel discovery and every scientist feels valued and supported.

**What changes do you think could improve the professional lives of scientists?**

Enhancing scientists' lives hinges on concrete measures: ensuring a living wage for focused research without financial distractions, fostering genuine mentorship for navigating scientific careers and providing access to the latest tools for innovation. Beyond resources, real commitment to diversity, equity and inclusion means transforming our workplaces into environments where diverse perspectives fuel discovery and every scientist feels valued and supported. It's about turning policy into practice, actively removing barriers, and creating a community where everyone's contribution is recognized for its unique value to science.

**What's next for you?**

My next step is to pursue an academic postdoctoral position, where I aim to advance our understanding of the role of neuronal circuits in shaping memory and social behaviors within the context of neurodevelopmental disorders. I'm particularly excited about the opportunity to explore how disruptions in these circuits contribute to behavioral deficits observed in neurodevelopmental disorders, including Rett syndrome. Ultimately, my goal is to contribute to the development of targeted interventions that can significantly improve the quality of life for individuals affected by these disorders.
